# Efficacy and Safety of Colchicine in Reducing Reperfusion Injury Among Patients With ST-Segment Elevation Myocardial Infarction (STEMI): A Systematic Review and Clinical Appraisal

**DOI:** 10.7759/cureus.91658

**Published:** 2025-09-05

**Authors:** Archana Dhami, Bassel Abdul Latif el Ejel, Mazhar Khalil, Sami Ul Haq, Mansi Yadav, Asma Iqbal, Syed Ali Ahsan, Helen O Uzokwe, Michelle C Uzokwe, Syed Faqeer Hussain Bokhari

**Affiliations:** 1 Family Medicine, Avalon University School of Medicine, Willemstad, CUW; 2 General Medicine, Stavropol State Medical University, Stavropol, RUS; 3 Medicine, Hayatabad Medical Complex Peshawar, Peshawar, PAK; 4 Medicine, Lahore General Hospital, Lahore, PAK; 5 Internal Medicine, Pandit Bhagwat Dayal Sharma Post Graduate Institute of Medical Sciences, Rohtak, IND; 6 Medicine and Surgery, Mayo Hospital, Lahore, PAK; 7 Medicine, King Edward Medical University, Lahore, PAK; 8 Family Medicine, Oceania University of Medicine, Apia, WSM; 9 Medicine, Columbus Central University, Ladyville, BLZ; 10 Surgery, King Edward Medical University, Lahore, PAK

**Keywords:** colchicine, inflammasome, ischemia-reperfusion, left ventricular thrombus, primary percutaneous coronary intervention, reperfusion injury, st-segment elevation myocardial infarction

## Abstract

This systematic review evaluated the efficacy and safety of colchicine in reducing ischemia-reperfusion injury among patients with ST-segment elevation myocardial infarction (STEMI) undergoing primary percutaneous coronary intervention. A comprehensive literature search across five databases identified 92 records, with five studies meeting the inclusion criteria, encompassing 390 patients. The included studies comprised randomized controlled trials and prospective studies investigating various colchicine dosing regimens, from short-term high-dose protocols to prolonged maintenance therapy. Primary outcomes included infarct size, inflammatory biomarkers, major adverse cardiovascular events, and safety profiles. Results demonstrated heterogeneous findings across studies. While one trial showed trends toward reduced inflammatory markers such as NLRP3 inflammasome activity, others failed to demonstrate significant reductions in reperfusion injury events or infarct size. Notably, the COVERT-MI trial revealed an unexpected threefold increase in left ventricular thrombus formation with colchicine therapy, raising safety concerns. Gastrointestinal side effects were also consistently reported across studies. The evidence suggests that the anti-inflammatory properties of colchicine may not translate into clinically meaningful cardioprotection in the acute phase of STEMI. Current data do not support routine colchicine administration for reperfusion injury reduction in STEMI patients undergoing primary percutaneous coronary intervention, highlighting the need for larger, well-designed trials with standardized protocols and longer follow-up periods to definitively establish its role in acute myocardial infarction management.

## Introduction and background

ST-segment elevation myocardial infarction (STEMI) remains a leading cause of morbidity and mortality worldwide, despite significant advances in timely diagnosis and reperfusion therapy [1]. Primary percutaneous coronary intervention (PCI) is the cornerstone of STEMI management and is pivotal in restoring coronary blood flow, thereby salvaging viable myocardium and improving clinical outcomes. However, while reperfusion is essential for limiting infarct size, it paradoxically initiates a cascade of deleterious cellular and molecular events collectively termed ischemia-reperfusion (I/R) injury. Reperfusion injury contributes significantly to final infarct size, impairs myocardial healing, and is associated with adverse left ventricular (LV) remodeling and heart failure [2,3]. Thus, there remains an unmet clinical need for adjunctive pharmacologic strategies that can mitigate myocardial I/R injury during acute STEMI.

I/R injury involves a complex interplay of mechanisms, including oxidative stress, calcium overload, mitochondrial dysfunction, microvascular obstruction, endothelial injury, and, most importantly, a robust sterile inflammatory response. Neutrophil infiltration, activation of the NLRP3 inflammasome, and release of pro-inflammatory cytokines such as interleukin-1β (IL-1β), IL-6, and tumor necrosis factor-α (TNF-α) are central to the pathogenesis of reperfusion injury [4,5]. These inflammatory mediators not only exacerbate cardiomyocyte necrosis and apoptosis but also promote adverse myocardial remodeling. Consequently, anti-inflammatory strategies have garnered increasing attention as potential therapies to complement reperfusion and protect the myocardium during STEMI.

Colchicine, a well-known anti-inflammatory agent derived from the *Colchicum autumnale* plant, has been used for centuries in the treatment of gout, familial Mediterranean fever, and pericarditis [6]. It exerts its anti-inflammatory effects by inhibiting microtubule polymerization, leading to the suppression of neutrophil activation, chemotaxis, and adhesion. Additionally, colchicine interferes with NLRP3 inflammasome assembly, thereby reducing IL-1β and IL-18 release [7]. These mechanisms provide a strong rationale for its potential utility in conditions characterized by excessive innate immune activation, including myocardial infarction and reperfusion injury [8].

Recent years have witnessed a resurgence of interest in colchicine as a cardioprotective agent in the context of coronary artery disease. Large-scale randomized controlled trials such as COLCOT (Colchicine Cardiovascular Outcomes Trial) and LoDoCo2 (Low-Dose Colchicine 2) have demonstrated significant reductions in recurrent cardiovascular events among patients with stable and unstable coronary syndromes, attributed primarily to the anti-inflammatory effects of colchicine [9,10]. In particular, the COLCOT trial included a subgroup of patients with recent myocardial infarction, showing favorable outcomes with low-dose colchicine initiated within 30 days of the event. These findings have spurred further investigation into the peri-infarct and acute-phase benefits of colchicine, especially its potential to reduce infarct size, improve myocardial salvage, and limit the progression to heart failure by attenuating reperfusion injury [9].

Experimental studies in animal models of myocardial infarction have shown that colchicine administered before or shortly after reperfusion can reduce infarct size, neutrophil infiltration, and inflammatory cytokine expression [11]. Mechanistic insights from preclinical studies support the role of colchicine in stabilizing endothelial function, reducing oxidative damage, and modulating the post-infarction inflammatory milieu. The role of colchicine in directly attenuating reperfusion injury in STEMI patients remains incompletely understood and variably studied. The timing of administration, optimal dosage, duration of therapy, and patient selection criteria are areas of ongoing exploration. Moreover, while inflammation is a key component of reperfusion injury, it is not the only pathophysiologic process involved, and the precise contribution of colchicine to myocardial protection during acute infarction requires critical appraisal. Variability in study designs, endpoints, and inflammatory markers across trials further complicates the ability to draw robust conclusions. This systematic review aims to synthesize the current body of evidence evaluating the efficacy and safety of colchicine in reducing reperfusion injury among patients with STEMI undergoing primary PCI. We seek to examine outcomes related to infarct size, LV function, inflammatory biomarker profiles, clinical endpoints (such as major adverse cardiovascular events), and adverse effects associated with colchicine use in the acute phase of myocardial infarction. By critically analyzing both preclinical and clinical studies, this review endeavors to clarify the therapeutic potential of colchicine as an adjunctive agent during STEMI reperfusion and to identify areas where further high-quality research is needed.

## Review

Materials and methods

This systematic review was conducted in accordance with the Preferred Reporting Items for Systematic Reviews and Meta-Analyses (PRISMA) 2020 guidelines to ensure methodological transparency and rigor throughout the processes of study identification, selection, eligibility screening, and data extraction [12].

Search Strategy

A comprehensive literature search was carried out across five major electronic databases, namely, PubMed, Scopus, Web of Science, EMBASE, and Hinari, from inception through April 2025. The search strategy incorporated a combination of Medical Subject Headings (MeSH) and free-text keywords to identify relevant studies assessing the role of colchicine in the management of reperfusion injury among patients with STEMI. Keywords used included "colchicine", "myocardial infarction", "STEMI", "reperfusion injury", "ischemia-reperfusion injury", "PCI", "primary PCI", "infarct size", "inflammatory response", and "cardioprotection". Boolean operators such as "AND" and "OR" were utilized to refine and combine the search terms appropriately. Each database was searched using tailored syntax to ensure optimal retrieval. Additionally, the reference lists of all included studies and relevant systematic reviews were manually screened to identify any studies that may have been missed during the initial database search.

Eligibility Criteria

We included peer-reviewed clinical studies that investigated the use of colchicine in patients diagnosed with STEMI undergoing primary PCI, specifically assessing its effect on I/R injury. Eligible study designs comprised randomized controlled trials, quasi-experimental studies, prospective or retrospective cohort studies, and case-control studies. Studies were required to report at least one relevant clinical or surrogate outcome, such as infarct size, LV function, troponin or CK-MB levels, inflammatory biomarkers (e.g., CRP and IL-6), myocardial salvage index, imaging findings (e.g., cardiac MRI), adverse events, or major adverse cardiovascular events (MACE). Exclusion criteria included animal studies, in vitro experiments, case reports, editorials, narrative reviews, conference abstracts, and articles not published in English. We also excluded studies where colchicine was used in contexts unrelated to myocardial reperfusion (e.g., chronic coronary syndrome or pericarditis).

Study Selection

All retrieved records were imported into the Rayyan software (Rayyan Systems Inc., Cambridge, Massachusetts, United States) for systematic de-duplication and blinded screening. Two independent reviewers screened titles and abstracts for potential relevance. Studies deemed eligible or requiring further clarification were subjected to full-text review by the same reviewers. Disagreements were resolved through discussion, and if consensus could not be reached, a third reviewer was consulted for arbitration. The entire selection process was documented using a PRISMA 2020 flow diagram, including the number of records screened, reasons for exclusion, and final studies included.

Data Extraction

Data extraction was independently performed by two reviewers using a predefined and piloted data extraction form. Extracted variables included first author, publication year, country, study design, sample size, timing of colchicine administration (pre-, peri-, or post-PCI), colchicine dosage and duration, and outcome measures such as infarct size, cardiac enzymes (e.g., troponin, CK-MB), imaging outcomes, inflammatory markers, adverse events, and clinical endpoints. Both baseline and post-intervention values were recorded where available. Any discrepancies in extracted data were resolved through consensus discussion or referred to a third reviewer when necessary.

Data Synthesis

Given the anticipated heterogeneity in study design, colchicine dosing regimens, timing of administration, and outcome assessment, a quantitative meta-analysis was not preplanned. Instead, a qualitative narrative synthesis was undertaken. Extracted outcomes were summarized in tabular format. Key trends, consistencies, and discrepancies across studies were described, and where applicable, subgroup observations (e.g., timing of colchicine initiation or variation in dosage) were noted. The limitations of individual studies and gaps in the current literature were also highlighted to inform directions for future research.

Results

Study Selection

**Figure 1:**
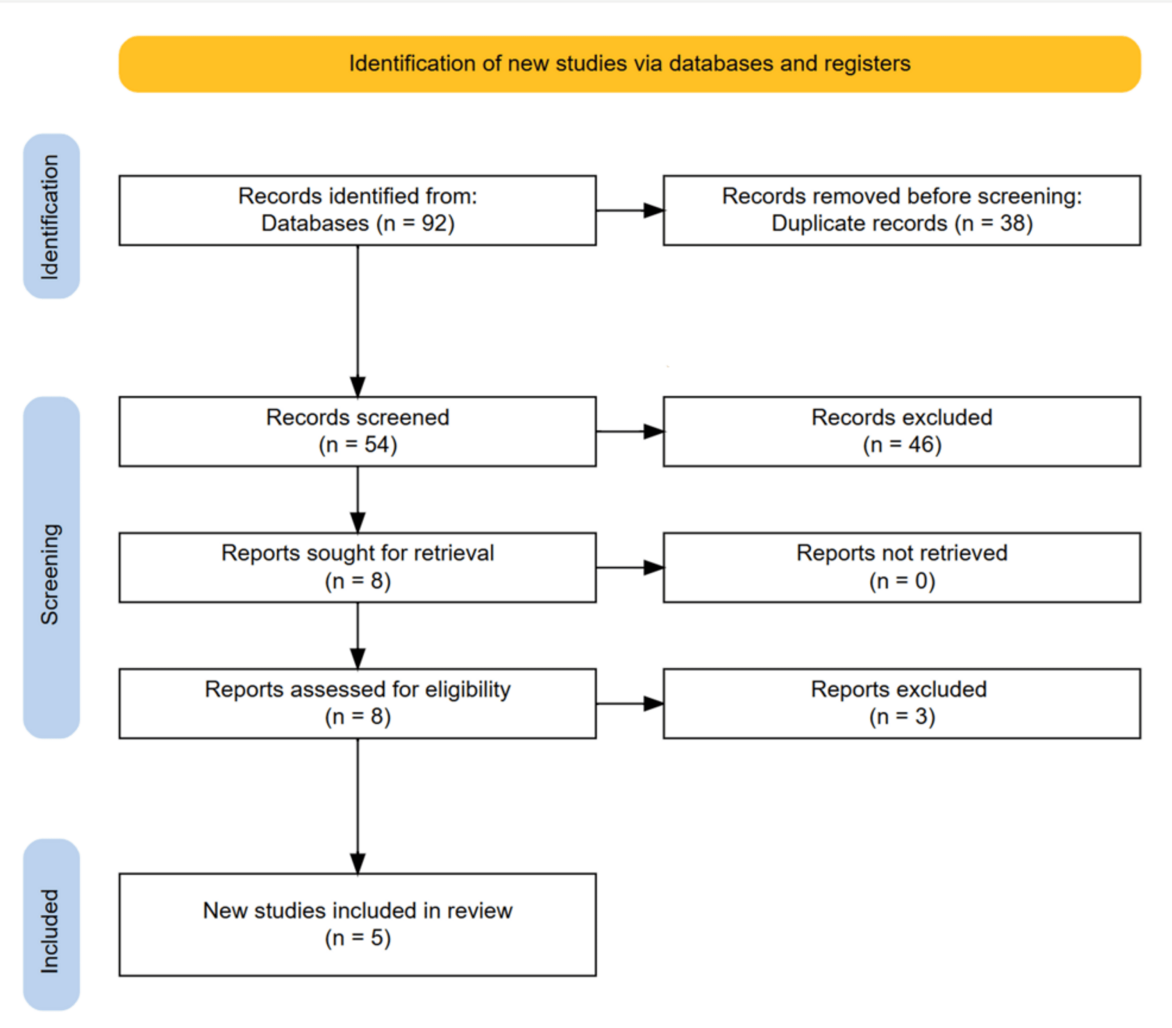
PRISMA diagram showing the study selection process PRISMA: Preferred Reporting Items for Systematic Reviews and Meta-Analyses

The initial database search yielded a total of 92 records. After removal of 38 duplicates, 54 unique articles remained for title and abstract screening. Of these, 46 studies were excluded due to irrelevance, non-clinical study design, absence of colchicine intervention, or unrelated outcomes. The remaining eight articles were selected for full-text review. Upon detailed evaluation, three studies were excluded for reasons including insufficient data on reperfusion injury outcomes, unclear intervention protocols, or inappropriate study design (e.g., case reports). Finally, five studies met the inclusion criteria and were included in the qualitative synthesis. A detailed depiction of the study selection process is presented in the PRISMA flow diagram (Figure 1).

Study Characteristics

The five included studies comprised 390 patients with STEMI undergoing primary PCI. Study designs included randomized controlled trials, with sample sizes ranging from 44 to 192 participants. Patient populations were predominantly male (72.9-82.5%), with mean ages between 55 and 60 years across studies. Colchicine dosing regimens varied considerably between trials. Most studies employed a loading dose of 2 mg followed by a maintenance therapy of 0.5 mg twice daily, though the duration differed from two days to one month. The study by Akodad et al. utilized 1 mg daily for one month without a loading dose [13]. Control groups received a matching placebo or standard medical therapy alone. The left anterior descending artery was the most commonly affected vessel (48.6-60.9% of cases), followed by right coronary artery involvement. Baseline cardiovascular risk factors were well-balanced between groups, including diabetes (11.9-40.5%), hypertension (29.7-62.5%), dyslipidemia (28.7-72.5%), and current smoking (42.9-75%). Primary endpoints varied across studies, encompassing inflammatory markers (CRP, NLRP3), infarct size measurements via cardiac MRI, reperfusion injury events, and major adverse cardiovascular events at follow-up periods ranging from five days to one year (Table 1).

**Table 1:** Summary of the main findings of the included studies BMI: body mass index; BD: twice daily; BL: baseline; AP: after procedure; CAD: coronary artery disease; CKD: chronic kidney disease; CMR: cardiac magnetic resonance; CRP: C-reactive protein; EQ-5D: EuroQol 5-dimension questionnaire; LAD: left anterior descending artery; LCx: left circumflex artery; LV: left ventricular; LVT: left ventricular thrombus; MACE: major adverse cardiovascular events; MI: myocardial infarction; NLRP3: nucleotide-binding domain, leucine-rich repeat, and pyrin domain containing 3; PCI: percutaneous coronary intervention; PPCI: primary percutaneous coronary intervention; QOL: quality of life; RCA: right coronary artery; STEMI: ST-segment elevation myocardial infarction; TIMI: thrombolysis in myocardial infarction

Author	Year	Study design	Sample size	Study duration	Mean age	Gender distribution		Infarct location	Intervention group	Control group	Efficacy outcomes	Safety outcomes	Main findings	Conclusions
Karim et al. [14]	2025	Randomized, double-blind, placebo-controlled clinical trial	77 patients (37 colchicine, 40 placebo)	December 2022 to April 2023	55.2±9.9 years	76.6% male, 23.4% female	Smoking (71.42%), dyslipidemia (colchicine 59.4%, placebo 72.5%), hypertension (colchicine 62.1%, placebo 62.5%), diabetes (colchicine 40.5%, placebo 32.5%), obesity (colchicine 45.9%, placebo 57.5%), CKD (colchicine 2.7%, placebo 2.5%), CAD (colchicine 5.4%, placebo 2.5%)	LAD: 53.24%; RCA: 36.4%; LCx: 10.4%	2 mg colchicine loading dose+0.5 mg BD for 2 days	Amylum at equivalent dosing	Reperfusion injury events	Side effects: colchicine 21.6% vs. placebo 15%. Most common: diarrhea (16%), hematemesis (2.7%), melena (2.7%)	Colchicine failed to reduce reperfusion injury compared to placebo	Colchicine administration in STEMI patients undergoing PPCI failed to reduce reperfusion
Bouleti et al. [15]	2024	Randomized, multicenter, double-blinded, placebo-controlled trial (COVERT-MI)	192 patients (101 colchicine, 91 placebo)	1 year follow-up (mean follow-up: 345±96)	60±10 years	19.5% women (21/101 colchicine, 17/91 placebo)	BMI: 27.3±5.0 colchicine, 26.9±4.4 placebo; current smoking: 43.6% colchicine, 42.9% placebo; diabetes: 11.9% colchicine, 14.3% placebo; hypertension: 29.7% colchicine, 31.9% placebo; dyslipidemia: 28.7% colchicine, 37.4% placebo	LAD: 51.5% colchicine, 48.4% placebo; LCx/RCA: 47.4% colchicine, 47.3% placebo	2 mg colchicine loading dose followed by 0.5 mg BD for 5 days	Matching placebo regimen	Primary endpoint: Composite of MACEs at 1 year (all-cause death, acute coronary syndromes, heart failure events, ischemic strokes, sustained ventricular arrhythmias, acute kidney injury). Secondary: Individual components of MACE. QOL assessed by EQ-5D score	LVT incidence: 22.2% colchicine vs. 7.4% placebo (p=0.01). No statistical difference in ischemic strokes (3% vs. 2.2%; p=0.99) despite higher LVT in the colchicine group	No significant difference in composite MACE: 35.6% colchicine vs. 44.1% placebo (p=0.3). No significant difference in all-cause death: 4% colchicine vs. 3.3% placebo (p=0.99). Non-significant trend toward fewer heart failure events in the colchicine group: 11.9% vs. 19.8% placebo (p=0.20). No significant difference in QOL scores at 1 year: 75.8±15.7 vs. 72.7±16.2 (p=0.18)	Short-term high-dose colchicine at the time of reperfusion and for 5 days in acute STEMI did not reduce MACEs at 1 year. Despite the initial increase in LVT in the colchicine group, there was no excess of ischemic adverse events at 1 year. The study does not support short-term colchicine treatment at acute phase of MI to reduce infarct size and improve outcomes
Karim et al. [16]	2024	Randomized controlled trial	77 patients (37 colchicine, 40 placebo)	December 2022 to April 2023	55.3±10.01 (colchicine), 55.15±9.88 (placebo)	Male: 72.9% (colchicine), 80% (placebo). Female: 22.1% (colchicine), 20% (placebo)	Diabetes: 40.5% vs. 32.5%; dyslipidemia: 59.4% vs. 72.5%; hypertension: 62.1% vs. 62.5%; smoking: 67.6% vs. 75%; obesity: 45.9% vs. 57.5%; CKD: 2.7% vs. 2.5%; CAD: 5.4% vs. 2.5%; mean STEMI onset: 6.0±2.78 h vs. 7.19±3.34 h	LAD: 48.64% vs. 57.5%; LCx: 16.21% vs. 5%; RCA: 35.13% vs. 37.5%	2 mg colchicine loading dose followed by 0.5 mg BD every 12 h for 48 h+standard treatment	Placebo+standard treatment	NLRP3 levels (BL, AP, 24H)	Not reported	The colchicine arm showed decreasing trend in NLRP3 from baseline to 24 h (38.69 to 37.67), while the placebo arm showed increase (39.01 to 42.89), though not statistically significant	Colchicine addition to standard treatment of STEMI patients undergoing PCI reduces NLRP3 level despite being statistically insignificant
Mewton et al. [17]	2021	Double-blind, randomized, placebo-controlled, multicenter trial	192 patients (101 colchicine, 91 placebo)	From July 20, 2018, to July 28, 2020 (follow-up at 5 days and 3 months)	60.0±10.5 years	19.5% female	First episode of STEMI referred for PPCI, occluded infarct-related artery (TIMI flow ≤1), presentation within 12 h of chest pain onset	Balanced between anterior and non-anterior territories	2 mg colchicine loading dose followed by 0.5 mg BD for 5 days	Matching placebo regimen	Primary: Infarct size (IS) by CMR at 5 days. Secondary: LV ejection fraction, microvascular obstruction mass, LV remodeling, IS at 3 months	Gastrointestinal side effects, LV thrombus frequency, major adverse cardiovascular events	No significant reduction in IS in the colchicine group (26 g (IQR 16-44) vs. 28.4 g (IQR 14-40); p=0.87). Unexpected 3-fold increase in LV thrombus in the colchicine group (22.2% vs. 7.4%; p=0.01). Higher incidence of gastrointestinal side effects with colchicine (34% vs. 11%; p=0.0002)	High-dose colchicine given orally at the time of reperfusion for a short period did not reduce myocardial damage induced by ischemia-reperfusion. Further studies exploring the timing, pharmacokinetics, and dose response of colchicine and other anti-inflammatory agents are needed
Akodad et al. [13]	2017	Interventional, open-label, controlled, prospective study	44 (23 intervention, 21 control)	December 2014 to May 2015 with one-month follow-up	60.1±13.1 (colchicine), 59.7±11.4 (control)	82.5% male (colchicine), 76.2% male (control)	Hypertension: 39.1% vs. 47.6%; diabetes: 13% vs. 14.3%; smoker: 73.9% vs. 66.7%; dyslipidemia: 34.8% vs. 38.1%	LAD: 60.9% vs. 33.3%; LCx: 13% vs. 9.6%; RCA: 26.1% vs. 57.1%	Colchicine 1 mg daily for 1 month+optimal medical treatment	Optimal medical treatment	Primary: CRP peak value. Secondary: troponin peak, hospitalization duration, MACE, cardiac remodeling	Digestive intolerance (43.4%), treatment discontinuation (13%); no hepatotoxicity or myelotoxicity	No significant difference in CRP peak (29.03 vs. 21.86 mg/L; p=0.36). No difference in other inflammatory markers or cardiac function measures	The effect of colchicine on inflammation in STEMI patients could not be demonstrated; further, larger studies are needed

Quality Assessment

The methodological quality of the included studies was assessed using the Newcastle-Ottawa Scale (NOS) for cohort studies and a modified version for randomized controlled trials. The five included studies demonstrated variable quality scores ranging from 6 to 8 out of 9 possible points. The randomized controlled trials (Bouleti et al., Mewton et al., and Karim et al.) scored higher due to their robust study designs, adequate randomization methods, and appropriate control groups [15-17]. However, limitations were noted across studies, including small sample sizes, short follow-up periods, and potential selection bias. The prospective study by Akodad et al. received a lower score due to its open-label design and lack of blinding [13]. Overall, the evidence base demonstrates moderate quality with inherent limitations affecting the strength of conclusions (Table 2).

**Table 2:** Quality assessment of the included studies using the Newcastle-Ottawa Scale (NOS). Stars (★) indicate points awarded for each domain: Selection (maximum 4 stars), Comparability (maximum 2 stars), and Outcome (maximum 3 stars). Total scores range from 0 to 9, with higher scores indicating better methodological quality

Author	Randomization	Allocation concealment	Blinding	Baseline comparability	Outcome assessment	Follow-up completeness	Sample size adequacy	Total score	Quality
Karim et al. [14]	★	★	★	★	★	☆	☆	6/9	Moderate
Bouleti et al. [15]	★	★	★	★	★	★	★	7/9	Good
Karim et al. [16]	★	★	★	★	★	☆	☆	6/9	Moderate
Mewton et al. [17]	★	★	★	★	★	★	★	7/9	Good
Akodad et al. [13]	★	☆	☆	★	★	☆	☆	5/9	Moderate

Discussion

The pathophysiological rationale for colchicine use in STEMI is compelling. I/R injury involves a cascade of inflammatory processes, including neutrophil infiltration, NLRP3 inflammasome activation, and cytokine release, all of which contribute to the final infarct size and adverse remodeling. The well-established anti-inflammatory mechanisms of colchicine, particularly its ability to inhibit microtubule polymerization and suppress inflammasome activity, provide a strong theoretical foundation for its cardioprotective potential. This rationale is further supported by successful outcomes in secondary prevention trials such as COLCOT and LoDoCo2, which demonstrated significant reductions in recurrent cardiovascular events through anti-inflammatory pathways [9,10].

However, the clinical reality presented in this review reveals a more nuanced picture. The five included studies demonstrate considerable heterogeneity in study design, dosing regimens, timing of administration, and outcome measures, making direct comparisons challenging. Most notably, the larger, well-designed trials failed to demonstrate significant benefits in primary endpoints. The COVERT-MI trial, the largest included study with 192 patients, showed no significant difference in the composite MACE endpoint at one year, despite employing a robust short-term high-dose protocol followed by maintenance therapy [14,16].

Perhaps most concerning is the unexpected safety signal observed in multiple studies. The threefold increase in LV thrombus formation reported in the COVERT-MI trial represents a significant and unexpected finding that warrants serious consideration [14,16]. While the mechanism underlying this observation remains unclear, it raises important questions about the effects of colchicine on coagulation pathways and endothelial function in the acute setting. This finding is particularly troubling given that LV thrombus formation is associated with increased risk of systemic embolization and stroke. The gastrointestinal side effects consistently reported across studies, affecting 11-43% of patients, represent another important consideration [13,15,17]. While generally mild and reversible, these effects occurred at rates substantially higher than those typically observed in chronic colchicine therapy, possibly reflecting the acute inflammatory state and altered drug metabolism in STEMI patients. The high incidence of digestive intolerance may limit clinical applicability and patient compliance, particularly in the vulnerable acute phase following myocardial infarction.

The timing of colchicine administration emerges as a critical factor that remains inadequately explored. While some studies initiated therapy before or during PCI, others began treatment hours after reperfusion. The narrow therapeutic window for interventions targeting reperfusion injury suggests that optimal timing may be crucial for efficacy. The inflammatory cascade initiated by reperfusion occurs within minutes to hours, and delayed intervention may miss the critical period for meaningful cardioprotection. Dosing strategy represents another area of uncertainty. The included studies employed various regimens, from single high-dose protocols to extended maintenance therapy. The optimal dose-response relationship for acute cardioprotection may differ significantly from that established for chronic anti-inflammatory effects in stable coronary disease. Higher doses may be required to achieve adequate tissue concentrations during the acute inflammatory phase, but this must be balanced against increased toxicity risk [18].

The heterogeneity in outcome measures across studies further complicates interpretation. While some studies focused on surrogate endpoints such as inflammatory biomarkers or infarct size by imaging, others examined clinical outcomes. The disconnect between anti-inflammatory effects and clinical benefits suggests that inflammation may not be the predominant mechanism driving reperfusion injury or that colchicine's anti-inflammatory effects may be insufficient to overcome other pathophysiological processes, such as calcium overload, oxidative stress, and microvascular dysfunction. The implications of these findings extend beyond colchicine specifically to the broader field of cardioprotection in STEMI. The failure of numerous promising cardioprotective agents to demonstrate clinical benefit despite strong preclinical evidence highlights the complexity of translating mechanistic insights into therapeutic success. The multifactorial nature of reperfusion injury may require combination therapeutic approaches rather than single-agent interventions.

Limitations and future directions

Several limitations constrain the interpretation of this systematic review. The small number of included studies and relatively modest sample sizes limit statistical power and generalizability. Significant heterogeneity in study designs, dosing protocols, timing of administration, and outcome measures precluded meaningful meta-analysis and quantitative synthesis. The predominance of single-center studies and varying follow-up periods further limit the robustness of conclusions. Future research should prioritize large, multicenter randomized controlled trials with standardized protocols addressing optimal dosing, timing, and duration of colchicine therapy. Mechanistic studies investigating the unexpected LV thrombus formation are urgently needed to inform safety profiles. Pharmacokinetic studies in acute STEMI patients may reveal altered drug disposition affecting efficacy. Investigation of combination therapies targeting multiple reperfusion injury pathways, biomarker-guided patient selection strategies, and longer-term follow-up examining heart failure progression and quality of life outcomes represent important research directions. Additionally, exploration of alternative anti-inflammatory agents with different mechanistic profiles may provide insights into the role of inflammation in reperfusion injury and identify more effective therapeutic targets.

## Conclusions

Current evidence does not support the routine use of colchicine for reducing I/R injury in STEMI patients undergoing primary PCI. Despite compelling mechanistic rationale and success in secondary prevention, the available clinical data demonstrate inconsistent efficacy and concerning safety signals, particularly increased LV thrombus formation. The heterogeneity in study designs and outcomes limits definitive conclusions, but the lack of consistent clinical benefit across multiple trials is noteworthy. While the anti-inflammatory properties of colchicine may modulate certain biomarkers, this does not translate into meaningful cardioprotection or improved clinical outcomes in the acute setting. The high incidence of gastrointestinal side effects further limits its clinical applicability. Until larger, well-designed trials with standardized protocols definitively establish both efficacy and safety, colchicine should not be considered standard therapy for reperfusion injury prevention in STEMI patients. Future research should focus on understanding the mechanisms underlying observed safety signals and identifying optimal patient selection criteria, dosing strategies, and combination approaches for cardioprotection in acute myocardial infarction.
